# Letter to the editor: Seasonality and effects of climatic exposures on community-acquired Legionnaires’ disease incidence, Italy, 2005 to 2023

**DOI:** 10.2807/1560-7917.ES.2026.31.29.2600567

**Published:** 2026-07-23

**Authors:** Giancarlo Ceccarelli, Fabio Scarpa, Cecilia Ceccarelli, Massimo Ciccozzi, Francesco Branda

**Affiliations:** 1 Department of Public Health and Infectious Diseases, University of Rome Sapienza, Rome, Italy; 2 Azienda Ospedaliero Universitaria Umberto I, Rome, Italy; 3 Genomics, AI, Bioinformatics, Infectious Diseases, Epidemiology Group (GABIE), Rome, Italy; 6 Unit of Medical Statistics and Molecular Epidemiology, Università Campus Bio-Medico di Roma, Rome, Italy; 4 Department of Biomedical Sciences, University of Sassari, Sassari, Italy; 5 Architectural Science School, Department of Architecture, “Roma Tre” University, Rome, Italy

**Keywords:** Legionella, Legionnaires’ disease, climate, temperature, rainfall, humidity, engineered water systems

**To the Editor:** Sciurti et al. reported compelling evidence that temperature, humidity and precipitation are associated with the incidence of community-acquired Legionnaires’ disease (LD) in Italy, identifying distinct temporal lag patterns between climatic exposures and disease occurrence [1]. Their study substantially advances our understanding of the climatic determinants of LD. However, the biological mechanisms underlying these delayed associations deserve further consideration.

Unlike many climate-sensitive infectious diseases, human infection with *Legionella pneumophila* is not directly driven by meteorological conditions. Rather, transmission depends on bacterial amplification within engineered water systems, where *Legionella* persists in multispecies biofilms, replicates intracellularly within free-living amoebae and interacts with diverse microbial communities before reaching concentrations capable of generating infectious aerosols [2-5]. Consequently, climatic variables are unlikely to act directly on disease occurrence. Instead, they are more likely to influence the ecological conditions governing bacterial persistence, amplification and dissemination within engineered aquatic environments.

Temperature affects bacterial replication, biofilm maturation and protozoan host activity, whereas precipitation and hydrological variability may alter water quality, nutrient availability, hydraulic conditions and disinfectant residuals, thereby modifying microbial community structure and ecosystem stability within building water systems [2-7]. Importantly, these ecological responses are inherently time-dependent. Biofilm development, microbial succession, i.e. the temporal replacement of microbial populations within the biofilm community, and host–microbe interactions evolve over days to weeks rather than immediately following environmental perturbations.

One biologically plausible interpretation is that the temporal lag described by Sciurti et al. [1] reflects the time required for climate-driven ecological changes within engineered water systems to generate conditions favouring *Legionella* amplification and subsequent human exposure. Thus, the interval between climatic variation and increased LD incidence may encompass not only the incubation period of infection but also the ecological processes preceding bacterial proliferation within the built water environment.

This interpretation is consistent with the growing recognition that drinking water systems function as complex microbial ecosystems rather than passive water distribution infrastructures [4-8]. The occurrence of opportunistic premise plumbing pathogens is shaped by interactions among biofilms, protozoan hosts, microbial community composition, hydraulic conditions and water management practices. Recent experimental evidence has further demonstrated that *Legionella* colonisation follows dynamic temporal trajectories during biofilm development, reinforcing the concept that microbial ecosystem dynamics are central determinants of pathogen persistence and amplification [5].

Considering engineered water systems as the ecological interface linking climatic variability to human infection also has practical implications. Predictive models based exclusively on meteorological variables may therefore capture only part of the mechanisms underlying LD seasonality. Integrating climatic information with indicators of the ecological status and vulnerability of engineered water systems, including microbiological surveillance, biofilm dynamics and system characteristics, may improve the prediction of periods at increased transmission risk and help explain why similar climatic conditions do not necessarily produce comparable epidemiological patterns.

Testing this interpretation will require studies integrating climatic observations with longitudinal environmental microbiology and ecological monitoring of engineered water systems. Such an approach could determine whether the epidemiological lag identified by Sciurti et al. [1] corresponds to measurable ecological transitions preceding increased human exposure, thereby strengthening the biological basis of climate-informed surveillance and prevention strategies for Legionnaires’ disease.
